# Thyroid Hormone Resistance Syndrome: From Molecular Mechanisms to Its Potential Contribution to Hypertension

**DOI:** 10.7759/cureus.49913

**Published:** 2023-12-04

**Authors:** Keerthana Prakash, Pousette Hamid

**Affiliations:** 1 Internal Medicine, California Institute of Behavioral Neurosciences and Psychology, Fairfield, USA; 2 Neurology, California Institute of Behavioral Neurosciences and Psychology, Fairfield, USA

**Keywords:** vascular resistance, endocrine disorders, hypertension, hyperthyroidism, hypothyroidism, thyroid hormone receptor mutation, inherited disorder, thyroid hormone insensitivity, thyroid hormone resistance

## Abstract

Thyroid hormone resistance (THR) is a rare inherited disorder that affects approximately one in every 40,000 live births. This condition arises from a mutation in the thyroid hormone receptor β, leading to reduced responsiveness of target tissues. It can result in a combination of hypothyroidism and hyperthyroidism symptoms in different tissues. The thyroid hormone is crucial for controlling blood pressure, and even small changes in its levels can have an effect on vascular resistance, cardiac performance, and heart rhythm. Both hypo- and hyperthyroidism have been associated with elevated blood pressure, underscoring the significant link between thyroid hormone sensitivity and vascular function. Considering thyroid hormone sensitivity is essential in clinical practice, particularly when managing patients with hypertension, to ensure personalized and effective treatment approaches. Monitoring thyroid function is essential during the diagnosis of hypertension, as thyroid dysfunction can often be corrected to normalize blood pressure. It's crucial to distinguish between essential hypertension and hypertension associated with a thyroid abnormality in THR. The mechanisms behind the development of hypertension in THR include reduced nitric oxide production, dysregulation of the renin-angiotensin-aldosterone system, impaired endothelial function, and mutations in the deiodinases. Physicians should understand the underlying mechanisms of THR and identify new therapeutic targets for hypertension in THR.

## Introduction and background

The syndrome of thyroid hormone resistance (THR), often referred to as the syndrome of impaired sensitivity to thyroid hormone (ISTH), is a condition marked by a reduction in the responsiveness of target tissues (the pituitary gland and/or peripheral tissues) to thyroid hormone (TH) [1,2]. It is a rare genetic disorder inherited as an autosomal dominant or recessive trait that affects one in 40,000 live births [3]. The THR syndrome is characterized by elevated levels of free triiodothyronine (fT3) and serum-free thyroxine (fT4), associated with non-suppressed thyroid-stimulating hormone (TSH) [4,5]. The mutation of the TH receptor β is the main cause of THR [6].

TH abnormalities have a significant impact on the cardiovascular system [7]. Although primary hypertension affects the majority of people with hypertension, secondary hypertension affects 10-15% of patients. About 10% of all patient instances of hypertension are explained by endocrine abnormalities, and about 1% of cases of hypertension are caused by thyroid disorders [8]. Thyroid disorders, such as hypo- or hyperthyroidism, can heighten the likelihood of developing hypertension [9,10]. This is because THs have a crucial role in controlling blood pressure (BP). Hence, THR has a positive correlation with systemic vascular resistance, systolic blood pressure (SBP), and diastolic blood pressure (DBP) [11].

Monitoring thyroid function is crucial throughout the workup for hypertension since, in the majority of cases, thyroid dysfunction can be corrected to normalize BP. That is the reason why it is important for clinicians to understand the pathophysiology of various thyroid disorders and to determine when further testing and treatment may be necessary. Thus, the purpose of this review is to present a summary of the significance of TH in the control of BP and the development of hypertension in THR. It also aims to explore the molecular mechanisms involved in THR and how they contribute to these conditions.

## Review

Mechanisms of THR

**Figure 1:**
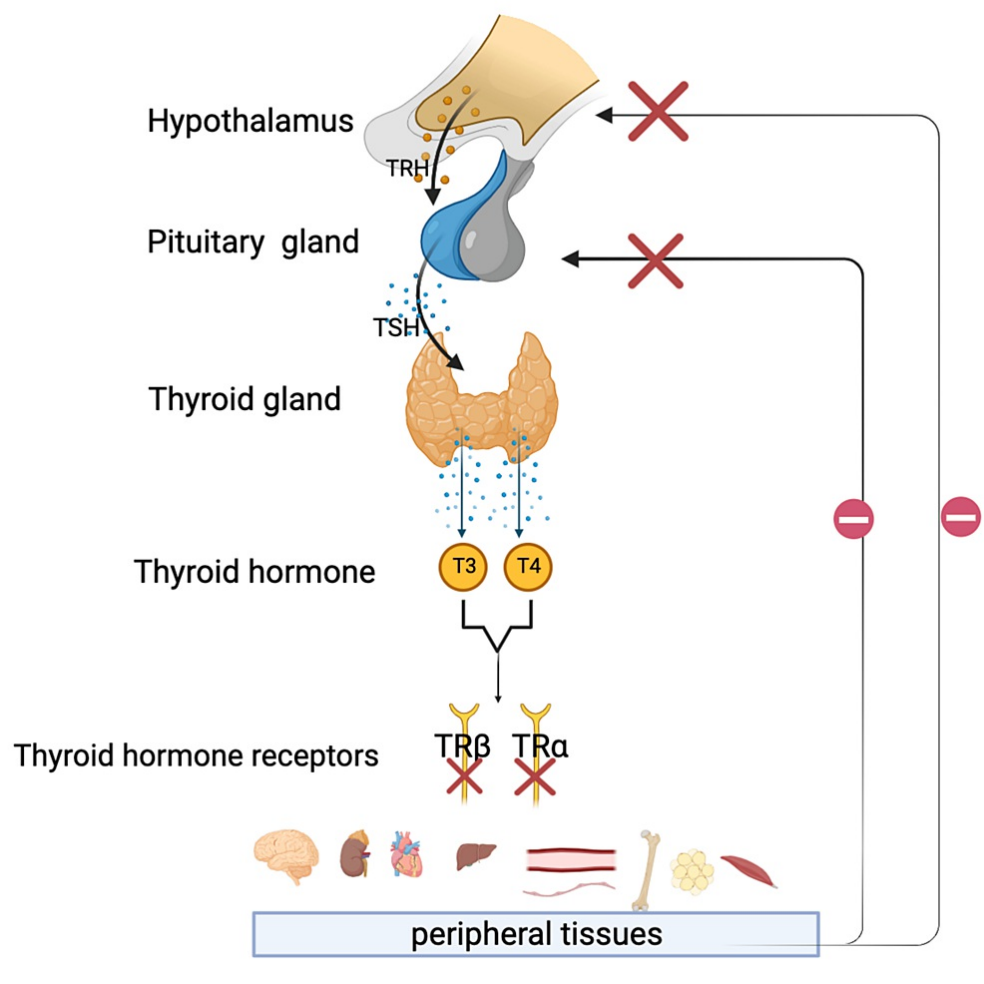
Thyroid hormone homeostasis: the HPT axis and feedback regulation HPT: hypothalamic-pituitary-thyroid; TSH: thyroid-stimulating hormone; TRH: thyrotrophin-releasing hormone; T3: triiodothyronine; T4: thyroxine; TRα: thyroid hormone receptor α; TRβ: thyroid hormone receptor β

THs, one of the most prevalent primary hormones in the body, play a crucial regulatory function in maintaining human health, including bone growth, tissue differentiation, brain development, cardiovascular homeostasis, and glycolipid metabolism. The thyroid gland synthesizes thyroxine (T4), the most prevalent type of TH, which circulates in the blood with low thyroid activity, while triiodothyronine (T3) is the most active form and is produced from T4 via deiodination in peripheral cells. A negative feedback mechanism in the body mediates the homeostasis of THs. The production of THs is regulated by the hypothalamic-pituitary-thyroid (HPT) axis, wherein the hypothalamus releases thyrotropin-releasing hormone (TRH) and the anterior pituitary gland secretes TSH. These hormones collectively control the synthesis and release of TH in the body. The thyroid hormone receptor β2 (TRβ2), which in turn gives feedback to the pituitary and hypothalamus to control the synthesis and secretion of TSH and TRH, also affects the concentrations of THs, as can be seen in Figure 1.

The two thyroid hormone receptor (TR) isoforms, thyroid hormone receptor alpha (TRα) and thyroid hormone receptor beta (TRβ), which are encoded by the genes THRA and THRB, respectively, are the targets of the action of THs [6]. These genes are located on different chromosomes, with THRA located on chromosome 17 and THRB located on chromosome 3 [12]. These isoforms are composed of various subtypes, such as TRα1, TRα2, TRβ1, and TRβ2. Several receptor isoforms appear to have different functions and distributions throughout the body, in addition to changing at various developmental stages. TRβ1 is the predominant isoform in the brain, liver, and kidneys; TRβ2 is primarily expressed in the hypothalamus, pituitary, inner ear, and retina and TRα1 in bone, skeletal, and cardiac muscles; and TR2 is widely expressed throughout the body [13,14]. TRα1 is highly expressed in the heart, bone, and skeletal muscle, whereas TRα2 is widely expressed throughout the whole body.

Resistance can occur at the level of the hypothalamus and pituitary gland, which leads to elevated levels of TSH. The elevated TSH levels stimulate the thyroid gland to increase the production of TH. However, the reduced action of TH in other tissues leads to compensatory hyporesponsiveness. The degree of hyporesponsiveness depends on the predominant TR isoform in the tissue, α or β [15].

After synthesis in the thyroid gland, these hormones are released into the bloodstream and delivered to target tissues throughout the body [16]. In order to enter target cells, THs use specific transporters, such as monocarboxylate transporters (MCTs) like MCT8, MCT10, and organic anion-transporting polypeptide 1C1 (OATP1C1), which facilitate their transport across the cell membrane [17]. Once inside the cell, THs are metabolized by a subfamily of selenoprotein enzymes called iodothyronine deiodinases [18]. They play a critical role in regulating TH activity and availability in target tissues. The amount of T3 accessible for receptor binding is determined by intracellular deiodinases, which include type 1 iodothyronine deiodinase (DIO1), type 2 iodothyronine deiodinase (DIO2), and type 3 iodothyronine deiodinase (DIO3).

DIO1 and DIO2 are responsible for the conversion of T4 to T3, whereas DIO1 and DIO3 catalyze the inactivation of T3 to reverse T3 (rT3) [15]. It is considered that the conversion of T4 to T3 by DIO2 is the primary mechanism for the synthesis of active THs, while inactivation of T4 and T3 by DIO1 and DIO3 regulates TH levels and activity. The balance between the activation and inactivation of THs by the deiodinases is critical for maintaining proper TH signaling and physiological function. Dysregulation of deiodinase activity can lead to various disorders, including hypothyroidism, hyperthyroidism, and resistance to TH.

**Figure 2:**
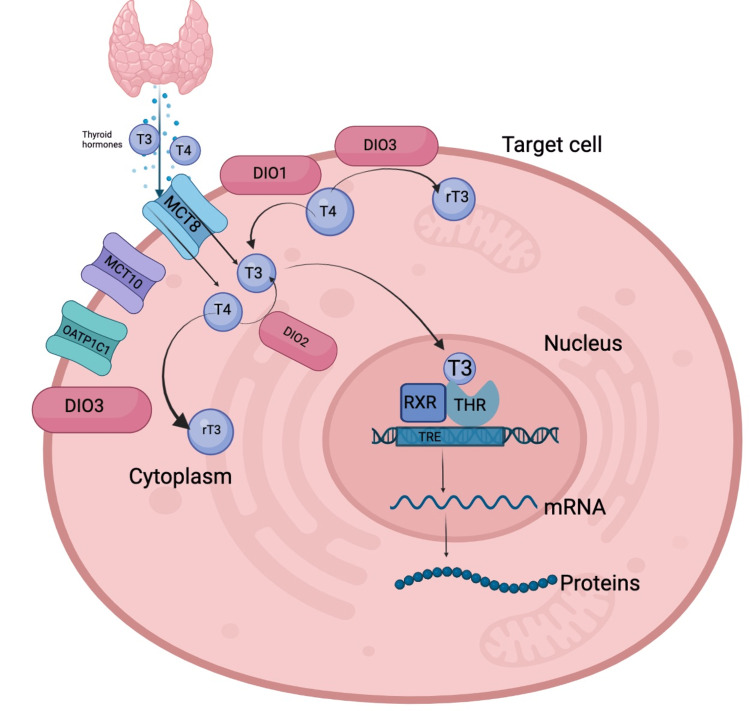
TH transport, metabolism, and signaling: factors to consider in TH regulation and cellular function TH: thyroid hormone; MCT8, MCT10, and OATP1C1: monocarboxylate transporters; DIO1, DIO2, and DIO3: intracellular deiodinases; RXR: retinoid X receptor; THR: thyroid hormone receptors; TRE: thyroid response elements; T3: triiodothyronine; T4: thyroxine; rT3: reverse T3

The factors related to TH signaling are mediated by T3 binding to its nuclear receptor (the TR), which functions as a ligand-dependent transcription factor to control the expression of target genes [19]. TRs are proteins that recognize specific DNA domains called thyroid response elements (TREs) in the regulatory regions of target genes. TRs can bind to TREs and activate or inhibit the expression of the associated genes, depending on the specific TRE sequence and the presence of other co-regulatory factors. To bind to TREs, TRs need to form homodimers or heterodimers with other nuclear receptors, such as the retinoid X receptor (RXR). This dimerization allows the receptor complex to recognize and bind to the specific DNA sequence of the TRE. Once bound to the TRE, TRs can recruit co-regulatory proteins that can either enhance or repress the expression of the associated gene shown in Figure 2 [16].

The HPT axis controls feedback mechanisms that keep circulating levels of TH within the reference range. The ability and availability of TH transporters (such as MCT8, MCT10, and OATP1C1), deiodinases (DIO1, DIO2, and DIO3), and nuclear TH receptors (TR1, TR1, and TR2) are required for optimal TH activity at targeted tissues [20]. THR is caused by defects in the TRs, deiodinases, and transporter proteins, which also include conditions that affect TH transport, biological activity, action, or metabolism. These conditions give rise to various clinical syndromes that are collectively referred to as disorders of TH signaling or resistance to TH syndromes, which are characterized by variable tissue hyporesponsiveness to TH [15,21].

ISTH can be classified as a TH cell transport defect, which occurs when the cell membrane hormone transport proteins are altered, or a TH metabolism defect, which occurs when there is an abnormal conversion of T4 into T3 that is not physiologically active. THR is another cause of ISTH [19]. The majority of instances include mutations in the TRβ, and only a limited number are caused by mutations in the TRα or other factors [22].

Clinical features

THR is a highly variable condition that can manifest with a wide range of symptoms, making it a phenotypically heterogeneous disease. Clinical manifestations can vary among individuals with identical genetic mutations. Goiter is the most common symptom, while sinus tachycardia, short stature, developmental delays, hair loss, ear infections, and abnormalities in the nose, throat, and psychological function are also frequently observed. In children, growth delays, delayed bone maturation, and cognitive impairments, along with hyperactivity and tachycardia, may coexist [23]. THR can cause both hypothyroidism and hyperthyroidism in different tissues, depending on which TRs are most prevalent in each organ, which could be TRβ (liver, adipose tissues) or TRα (skeletal and cardiac muscles, vascular endothelium) [24]. Recent research suggests that some people with euthyroid levels may exhibit symptoms of hypothyroidism due to reduced sensitivity to TH [25].

The role of THs in BP regulation

The thyroid gland and the cardiovascular system have a close and functional relationship, as they share the same embryonic origin and influence the normal function of the heart's components. For this reason, patients with cardiovascular diseases often undergo thyroid function tests to rule out TH imbalances. Even minor changes in TH levels can affect vascular resistance, cardiac contractility, BP, and heart rhythm, as TRs are present in these tissues and are sensitive to changes in circulating TH levels [26].

THs have a significant impact on vascular function, with both T3 and T4 acting as vasodilators on vascular smooth muscle cells [27]. In euthyroid individuals, an increase in TH concentration may serve as a compensatory mechanism for high BP. Hypo- and hyperthyroidism have been associated with elevated BP, which could be attributed to the genomic or non-genomic effects of THs on the vascular system and heart [28].

The mechanism underlying the impact of THs on vascular function involves the stimulation of nitric oxide (NO), which is an important endothelium-derived relaxing factor (EDRF), and the regulation of local regulatory factors, leading to a decrease in vascular smooth muscle tone [29,30,31,32]. NO plays a crucial role in maintaining vascular tone and regulating BP by promoting vasodilation and improving blood flow. This effect positively influences endothelial integrity and serves as a regulator of the renin-angiotensin-aldosterone system, which is involved in cardiovascular remodeling, atherosclerosis, heart failure, and BP control [33]. THs, especially T3, have been found to enhance the synthesis and release of renin as well as the production of angiotensin II in the liver. This leads to the increased expression of angiotensin-converting enzyme (ACE) and higher levels of angiotensin II, which can contribute to BP regulation [34].

Mechanisms underlying hypertension in THR

Abnormal results in thyroid function tests are often indicative of THR in clinical settings. Elevated serum levels of fT4 and/or fT3 and the absence of suppressed TSH levels are the key indicators of this condition [1]. Studies have shown that euthyroid individuals with higher normal TSH levels are at a greater risk of developing hypertension than those with lower normal TSH levels [35].

A current topic of interest is the reduced sensitivity to TH, which has been identified as a more common finding in the general population, as well as a possible association between this condition and metabolic parameters. Impaired sensitivity to THs may increase the risk of hypertension, possibly due to changes in TH concentration and activity.

Additionally, it is believed that this condition may impact vascular function [36]. The clinical manifestations of THR syndrome show a wide range of symptoms that can fall between hypothyroidism and hyperthyroidism in different tissues. This suggests that the organ's primary receptors, such as TRβ or TRα, could result in a combination of TH deficiency, sufficiency, and excess in the same patient [24].

Hyperthyroidism can lead to hyperdynamic circulation, characterized by increased heart rate, cardiac contractility, and erythropoietin synthesis. Additionally, these hormones can decrease peripheral vascular resistance, thereby contributing to an elevated SBP [37]. Elevated levels of renin synthesis and secretion are directly or indirectly stimulated by T3, and in hyperthyroidism, other hormonal factors associated with hypertension, such as endothelin-1, were also found to be increased [8].

In contrast, hypothyroidism is linked to elevated peripheral vascular resistance due to reduced levels of EDRF, which cause the contraction of vascular endothelial cells [38]. The production of NO is reduced, leading to decreased endothelium-dependent vasodilation and increased peripheral vascular resistance. This can result in hypertension and other cardiovascular complications. It may also cause hyperlipidemia and decreased renin secretion [39]. Patients with hypothyroidism also tend to have higher DBP levels [40].

**Figure 3:**
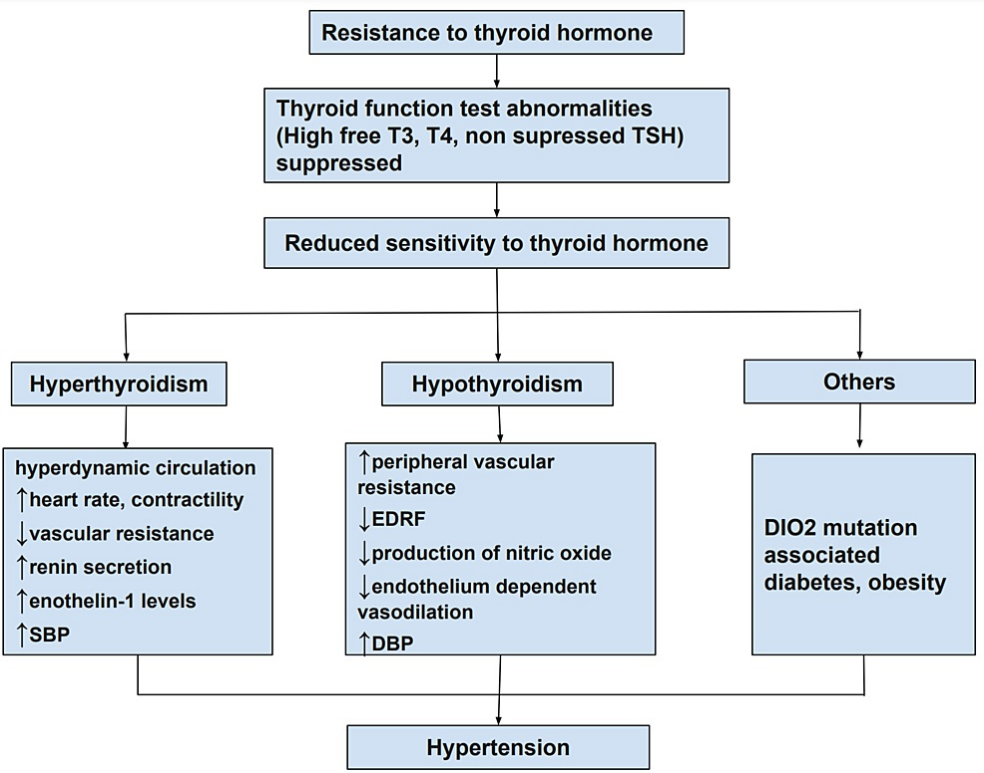
Mechanisms underlying hypertension in THR THR: thyroid hormone resistance; T3: triiodothyronine; T4: thyroxine; TSH: thyroid-stimulating hormone; SBP: systolic blood pressure; DBP: diastolic blood pressure; EDRF: endothelium-derived relaxing factor

Several studies have indicated that a mutation in the DIO2 gene can raise the risk of hypertension in individuals who are euthyroid [41]. The process of converting T4 to T3 may be hindered, resulting in reduced T3 levels and changes in the signaling of THs. DIO2 is crucial in regulating thyroid feedback in the pituitary gland, and reduced TH sensitivity due to impaired DIO2 expression may be linked to hypertension. Additionally, diabetes and obesity are also recognized risk factors for hypertension and have been found to be associated with impaired TH sensitivity [42,43]. These mechanisms underlying hypertension in THR are depicted in Figure 3.

Measures of THR and their association with hypertension

Compared to measuring serum hormone levels, assessing local hormone sensitivity may provide a more accurate representation of an individual's thyroid status. The regulation of TSH levels in the pituitary gland is influenced by TH through a negative feedback mechanism [36]. A new composite index called the Thyroid Feedback Quantile-Based Index (TFQI) has been proposed to reflect the pituitary's response to TH or central sensitivity to TH [42]. The TFQI has been shown to indicate reduced sensitivity to TH, which has been linked to an increased risk of hypertension [36].

The Total T4 Hormone Resistance Index (TT4RI) is a measure of THR that reflects the body's ability to respond to T4 levels. A high TT4RI value indicates reduced responsiveness to THs. The Thyroid-Stimulating Hormone Resistance Index (TSHI) is a measure of THR that reflects the body's ability to respond to TSH. A high TSHI value indicates reduced responsiveness to TSH, which can lead to decreased TH production and release.

According to the latest survey, there was a strong and consistent association found between high BP and all measures of THR, including TT4RI, TSHI, and TFQI, even in individuals who had normal thyroid function.

An elevation of the pituitary set point of the thyroid axis, as measured by the Pituitary Thyroid Feedback Quantitative Index (PTFQI), is associated with an increased prevalence of hypertension in euthyroid (normal thyroid function) adults. This means that individuals with higher PTFQI values may be at greater risk of developing high BP [44]. It was found that the TFQI and PTFQI were positively associated with hemodynamic parameters such as SBP, DBP, pulse pressure, mean arterial pressure, and rate-pressure product, as well as markers of arterial stiffness [39]. This finding may help to explain why reduced TH levels can lead to decreased arterial smooth muscle relaxation, resulting in increased systemic vascular resistance, reduced availability of NO, and reduced endothelium-dependent vasodilatation [33,38,27,44].

Management

The symptoms of THR are not specific and can be easily overlooked or misdiagnosed. Therefore, the detection of the THRB gene is crucial in confirming the diagnosis, especially for patients with high levels of fT3 and fT4, along with non-suppressed TSH, and their family members. Early diagnosis through gene sequencing can minimize the risk of adverse outcomes and is considered the standard approach for diagnosing THR [45].

Assessing thyroid function with TSH, fT4, and fT3 tests, BP monitoring over time and genetic testing, taking the patient's medical history into account, monitoring the patient's response to TH therapy, and working with specialists are all necessary to distinguish essential hypertension from that associated with thyroid abnormalities in THR.

The management objective for individuals with THR is to maintain a normal serum TSH level and achieve a balanced metabolic state. The treatment approach is tailored to the specific characteristics and symptoms of each patient. Those who are in a compensated euthyroid state typically do not require any treatment. However, individuals experiencing symptoms of hypothyroidism or hyperthyroidism may benefit from interventions such as TH supplementation, beta-blockers, anti-thyroid medications, or TH analogs. The choice of treatment depends on the specific features and circumstances of each patient [46]. However, normalization of serum T4 may not instantly result in the resolution of hypertension; hence, antihypertensive medications should also be used to treat moderate to severe hypertension [47].

Thyroid disorders like hyperthyroidism and hypothyroidism have been correlated with high BP. In addition to addressing the underlying thyroid dysfunction, proper treatment for certain thyroid-related disorders may possibly decrease hypertension. Furthermore, implementing lifestyle modifications aimed at promoting thyroid health, such as adopting a healthy diet, engaging in regular exercise, managing stress, and improving sleep quality, may also contribute to better BP control. It is crucial to monitor BP regularly to ensure its proper management and to assess the effectiveness of treatment and lifestyle interventions.

Effectively managing hypertension in patients with THR requires a personalized approach that takes various factors into consideration, such as thyroid function, cardiovascular health, and medication tolerance. Recognizing the strong connection between TH sensitivity and vascular function, it becomes essential to evaluate TH sensitivity when assessing thyroid status in clinical practice, especially among hypertensive patients [36]. This approach presents a promising opportunity to enhance hypertension prevention, screening, and the timely implementation of effective treatment strategies. Nonetheless, further research is warranted to determine the optimal management of cardiovascular complications in individuals with THR, as this area still lacks clarity.

## Conclusions

THR, characterized by impaired sensitivity to THs, can impact vascular function and contribute to hypertension. Patients with hyperthyroidism are associated with higher SBP, while hypothyroidism is linked to higher DBP. Elevated levels of fT3, fT4, and non-suppressed TSH are indicative of this condition. Early diagnosis through gene sequencing can help minimize adverse outcomes. The pathophysiology of hypertension in THR involves mechanisms such as reduced NO production, dysregulation of the renin-angiotensin-aldosterone system, impaired endothelial function, and DIO2 gene mutations. Various measures of THR, including TFQI, TT4RI, TSHI, and PTFQI, have shown a positive correlation with BP parameters. Treatment typically involves TH supplementation to overcome resistance and maintain normal hormone levels, which indirectly benefits hypertension. Antihypertensive drugs may be required for moderate to severe hypertension. Nevertheless, further research is needed to fully comprehend the underlying mechanisms and identify new therapeutic targets for hypertension in THR.
